# Nitric oxide‐enhanced Shiga toxin production was regulated by Fur and RecA in enterohemorrhagic *Escherichia coli* O157

**DOI:** 10.1002/mbo3.461

**Published:** 2017-03-15

**Authors:** Kimitoshi Ichimura, Takeshi Shimizu, Akio Matsumoto, Shinichiro Hirai, Eiji Yokoyama, Hiroki Takeuchi, Kinnosuke Yahiro, Masatoshi Noda

**Affiliations:** ^1^ Departments of Molecular Infectiology Graduate School of Medicine Chiba University Chiba Japan; ^2^ Pharmacology Graduate School of Medicine Chiba University Chiba Japan; ^3^ Division of Bacteriology Chiba Prefectural Institute of Public Health Chiba Japan

**Keywords:** enterohemorrhagic *Escherichia coli*, Fur, nitric oxide, NO reductase, RecA, Shiga toxin

## Abstract

Enterohemorrhagic *Escherichia coli* (EHEC) produces Shiga toxin 1 (Stx1) and Shiga toxin 2 (Stx2). Nitric oxide (NO), which acts as an antimicrobial defense molecule, was found to enhance the production of Stx1 and Stx2 in EHEC under anaerobic conditions. Although EHEC O157 has two types of anaerobic NO reductase genes, an intact *norV* and a deleted *norV*, in the deleted *norV*‐type EHEC, a high concentration of NO (12–29 μmol/L, maximum steady‐state concentration) is required for enhanced Stx1 production and a low concentration of NO (~12 μmol/L, maximum steady‐state concentration) is sufficient for enhanced Stx2 production under anaerobic conditions. These results suggested that different concentration thresholds of NO elicit a discrete set of Stx1 and Stx2 production pathways. Moreover, the enhancement of Shiga toxin production in the intact *norV*‐type EHEC required treatment with a higher concentration of NO than was required for enhancement of Shiga toxin production in the deleted *norV*‐type EHEC, suggesting that the specific NorV type plays an important role in the level of enhancement of Shiga toxin production in response to NO. Finally, Fur derepression and RecA activation in EHEC were shown to participate in the NO‐enhanced Stx1 and Stx2 production, respectively.

## 1 Introduction

Enterohemorrhagic *Escherichia coli* (EHEC) O157 is a causative agent of intestinal disorders ranging from mild infection to severe, bloody diarrhea (hemorrhagic colitis) (Hofmann, 1993; Keusch & Acheson, 1997; Lansbury & Ludlam, 1997). EHEC O157 associated with hemolytic‐uremic syndrome (HUS) has been shown to produce Shiga toxin 1 (Stx1) and Shiga toxin 2 (Stx2). Stx1, which is identical to the Shiga toxin produced by *Shigella dysenteriae* type 1 (Tesh & O'Brien, 1991), and Stx2 share 54% amino acids sequence homology (Jackson, Neill, O'Brien, Holmes, & Newland, 1987). Both Stx1 and Stx2 are members of the AB_5_ toxin family (Calderwood, Auclair, Donohue‐Rolfe, Keusch, & Mekalanos, 1987; De Grandis et al., 1987; Fraser et al., 2004; Jackson, Newland, Holmes, & O'Brien, 1987; Yutsudo, Nakabayashi, Hirayama, & Takeda, 1987). The A subunit is an RNA‐*N*‐glycosidase that plays a role in protein synthesis inhibition in eukaryotic cells (Endo et al., 1988). The pentamer of B subunits binds to the surface receptor, glycolipid receptor Gb3, on the target cells (Jacewicz, Clausen, Nudelman, Donohue‐Rolfe, & Keusch, 1986).

Both Shiga toxin genes in EHEC are located within Stx‐encoding phages that are related to the λ phage, which is well‐characterized with respect to both its genome arrangement and transcription patterns (Shimizu, Ohta, & Noda, 2009; Waldor & Friedman, 2005). However, the conditions under which they are highly expressed are different (Calderwood & Mekalanos, 1987; Calderwood et al., 1987; De Grandis et al., 1987; Hull, Acheson, Echeverria, Donohue‐Rolfe, & Keusch, 1993). Stx2 production is activated by phage‐inducing agents, such as mitomycin C, indicating that the *stx2* gene is transcribed from the phage late promoter (*P*_R_') of the Stx2‐encoding phage. Since the phage lysis genes of Stx2‐encoding phage as well as *stx2* are transcribed by the *P*_R_' during Stx2‐encoding phage induction, the Stx2‐encoding phage and Stx2 are released from bacterial cells at the same time (Shimizu et al., 2007, 2009). In contrast, the Stx1‐encoding phage in EHEC is less sensitive to phage‐inducing agents than the Stx2‐encoding phage, with the result that the level of Stx1 production induced by mitomycin C is lower. The Stx1‐encoding phage carrying the *stx1* contains the *stx1* promoter (*P*_Stx1_) region, which represents the functional operator‐binding site (Fur box) for the Fur (Calderwood & Mekalanos, 1987; Porcheron & Dozois, 2015). Fur is an iron‐responsive repressor of iron‐transport systems in *E. coli* (Lee & Helmann, 2007). Under low‐iron conditions, Stx1 production is increased (Calderwood & Mekalanos, 1987; Shimizu et al., 2009). Thus, there are two types of promoters for Stx1 expression in EHEC. Stx1 was mainly regulated by the *P*_Stx1_ and remained in bacterial cells (Shimizu et al., 2007, 2009).

Nitric oxide (NO) is a crucial cellular signaling molecule involved in many physiological and pathological events, e.g., cell cycle regulation, vascular relaxation, apoptosis, hypoxia, nutrient deficiency, and antimicrobial defense (Fang, 2004; Thomas et al., 2008). Because of the enormous variety of chemical reactions and biological properties associated with NO, the responses to this molecule are highly diverse. The maintenance of steady‐state NO concentrations has emerged as a key determinant of the biological function of NO. Precise cell responses are differentially regulated by the specific NO concentration. Though the synthesis and diffusion of NO are partial determinants of its concentration, consumption of NO is also important for determining its biological function. The rate of NO consumption by cells is directly dependent on the oxygen concentration (Thomas, Liu, Kantrow, & Lancaster, 2001), suggesting an important regulatory relationship between NO signaling and oxygen concentration in different environments. Whereas increased oxygen levels will increase NO consumption, NO consumption is decreased under anaerobic conditions.

In humans, NO is produced by the inducible isoform of NO‐synthase (iNOS) in several cell types, including macrophages, as part of the immune response to counteract microbial infection. NO induces a bacterial SOS response, which plays a central role in the *E. coli* response to a wide variety of genotoxic agents (Lobysheva, Stupakova, Mikoyan, Vasilieva, & Vanin, 1999; Schapiro, Libby, & Fang, 2003; Spek et al., 2001; Stupakova, Lobysheva, Mikoyan, Vanin, & Vasilieva, 2000). The SOS regulatory system, which is involved in the induction of more than 40 genes upon blockage of ongoing DNA replication, controls the response to DNA damage or the inhibition of DNA replication (Walker, 1984). DNA damage causes RecA polymerization around a single‐stranded DNA (Kowalczykowski, 2000). This active filament form can direct homologous recombination (Cox, 1999). Activated RecA in turn mediates the cleavage of LexA (Little, 1991), inactivating it and resulting in derepression of the SOS regulon (Fernandez De Henestrosa et al., 2000). Moreover, to perform specific roles in SOS mutagenesis, RecA also promotes UmuD cleavage (Pham et al., 2002). In addition, RecA promotes cleavage of the phage CI repressor, triggering induction of the lytic cycle (Little, 1984, 1993), and late gene expression by the Stx‐encoding prophage results from the Stx‐encoding prophage induction in EHEC (Wagner et al., 2002). Conversely, NO decreases *stx2* expression in EHEC O157 to repress the SOS response by the NO sensor nitrite‐sensitive repressor NsrR (Vareille, de Sablet, Hindre, Martin, & Gobert, 2007). Moreover, NO also inhibits the expression of the locus of enterocyte effacement (LEE) genes in EHEC (Branchu et al., 2014). NO is thus a signaling mediator with many diverse and often opposing biological activities in bacterial cells.

As a strategy to evade the host immune attack, pathogenic bacteria have evolved a biochemical pathway to degrade NO. *E. coli* has evolved several mechanisms for NO detoxification (Poole, 2005; Spiro, 2006, 2007). It expresses a flavorubredoxin (NorV), which reduces NO to N_2_O under anaerobic conditions (Gardner, Costantino, & Salzman, 1998; Gardner & Gardner, 2002; Gardner, Helmick, & Gardner, 2002; Spiro, 2012). It also expresses a flavohemoglobin (HmpA), and then utilizes O_2_ to convert NO to nitrate under aerobic conditions and reduces NO to N_2_O under anaerobic conditions (Householder, Fozo, Cardinale, & Clark, 2000; Kim, Orii, Lloyd, Hughes, & Poole, 1999; Poole, 2005; Watmough et al., 1999). However, the rate of NO reduction by HmpA is very low (Gardner & Gardner, 2002). Since NorV is sensitive to O_2_, NO reductase activity detoxifies NO under anaerobic conditions. Recent study suggested that the *E. coli* Hcp may be a high affinity NO reductase that is one of the enzymes used to reduce NO to N_2_O under anaerobic conditions (Wang et al., 2016). Since NorV, HmpA and Hcp in *E. coli* can detoxify NO throughout the physiological O_2_ range, *E. coli* is able to resist large amounts of NO. However, in EHEC O157, there are two types of *norV* genes, an intact *norV* and a 204 bp‐deleted *norV* (Gardner et al., 2002). The products of deleted *norV* did not exhibit NO reductase activity under anaerobic conditions (Shimizu, Tsutsuki, Matsumoto, Nakaya, & Noda, 2012). The intact *norV*‐type EHEC induced lower NO production and higher Stx2 production within macrophages than deleted *norV*‐type EHEC O157 (Shimizu et al., 2012). Therefore, intact *norV*‐type EHEC O157 showed a better level of survival than deleted *norV*‐type EHEC, suggesting that the intact *norV* was a direct virulence determinant of EHEC O157 under anaerobic conditions. Furthermore, an evolutionary analysis revealed that intact *norV* in EHEC O157 was strictly correlated with subgroup C cluster 1, and deleted *norV* was correlated with subgroup C clusters 2 and 3 (Shimizu, Hirai, Yokoyama, Ichimura, & Noda, 2015).

In this study, we examined the effect of NO on Shiga toxin production in EHEC O157 under anaerobic conditions. We elucidated the role of intact NorV in NO‐promoted Shiga toxin production. We also found that enhanced Stx1 and Stx2 production in response to NO was involved in Fur derepression and RecA activation in EHEC O157, respectively.

## 2 Materials and Methods

### 2.1 EHEC strains, plasmids, and oligonucleotides

The EHEC strains and plasmids used in this study are listed in Table 1. The oligonucleotides used for this study are shown in Table S1.

**Table 1:** Bacterial strains and plasmids

Strain or plasmid	Relevant characteristics	Reference
EDL933	EHEC O157, *stx1, stx2,* deleted *norV*	Perna et al. (2001)
E1‐E2S	EDL933, insertion of *luxE* in downstream of *stxB1*	Shimizu et al. (2011)
E(SR)2‐E1S	EDL933, insertion of *luxE* in downstream of *stxB2,* deletion of *S‐R* in Stx2‐phage	Shimizu et al. (2011)
ERSA	EDL933, insertion of *luxE* in downstream of *recA*	This study
EVm	EDL933, replacement of intact *norV*	Shimizu et al. (2012)
K2	EHEC O157, *stx1, stx2,* deleted *norV*	Shimizu et al. (2012)
K16	EHEC O157, *stx1, stx2,* intact *norV*	Shimizu et al. (2012)
K24	EHEC O157, *stx1,* deleted *norV*	Shimizu et al. (2012)
K42	EHEC O157, *stx2,* deleted *norV*	Shimizu et al. (2012)
K43	EHEC O157, *stx2,* intact *norV*	Shimizu et al. (2012)
K58	*E. coli* O157, intact *norV*	Shimizu et al. (2012)
K15	EHEC O157, *stx1, stx2,* intact *norV*	Shimizu et al. (2012)
K15(‐V)	K15, deletion of intact *norV*	Shimizu et al. (2012)
ERA1‐1	EDL933, deletion of *recA*	This study
ERG22Y	EDL933, *recA* (G22Y)	This study
ERG24Y	EDL933, *recA* (G24Y)	This study
ERE123A	EDL933, *recA* (E123A)	This study
ERG204S	EDL933, *recA* (G204S)	This study
ERD224A	EDL933, *recA* (D224A)	This study
ERG288Y	EDL933, *recA* (G288Y)	This study
EP	EDL933, deletion of *stx1* promoter	This study
EH	EDL933, deletion of *hmpA*	Shimizu et al. (2012)
EDLf	EDL933, deletion of *fur*	This study
E1Q1	EDL933, deletion of *Q* of Stx1‐encoding phage	This study
DH5α (λ*pir*)	*recA1, endA1, gyrA96, thi‐1, hsdR17, supE44, relA1, deoR, (lacZYA‐argF)U169, ⌊pir* ^*+*^	Elliott and Kaper (1997)
pRed/ET (amp)	Red/ET expression plasmid, Amp^r^	Gene Bridges GmbH
placlux8	LuxCDABE expression plasmid, Amp^r^, *P*_*lac*_	Shimizu et al. (2011)
pRPL3	NO reporter plasmid, *norR‐norV* promoter‐*luxCDABE* fusion, Amp^r^	Shimizu et al. (2012)
pLCE19	Suicide plasmid, *luxE* and *loxP*‐flanked Cm^r^ cassette	Shimizu et al. (2011)
pluxCDAB3	LuxCDAB expression plasmid, Amp^r^, *P*_*lac*_	Shimizu et al. (2011)
pluxStx1P2	*stx1* reporter plasmid, *stx1* promoter‐*luxCDABE* fusion, Amp^r^	This study
pluxStx1PGG6	Mutated *stx1* reporter plasmid, *stx1* promoter‐*luxCDABE* fusion, mutation of Fur box sequence, Amp^r^	This study
pFRT‐Kan	Suicide plasmid, FRT‐flanked PGK‐gb2 neo cassette	Gene Bridges GmbH
pFRT‐recA2	Suicide plasmid, *recA* and FRT‐flanked PGK‐gb2 neo cassette	This study
precAG22Y1	Suicide plasmid, *recA* (G22Y) and FRT‐flanked PGK‐gb2 neo cassette	This study
precAG24Y1	Suicide plasmid, *recA* (G24Y) and FRT‐flanked PGK‐gb2 neo cassette	This study
precAE123A3	Suicide plasmid, *recA* (E123A) and FRT‐flanked PGK‐gb2 neo cassette	This study
precAG204S3	Suicide plasmid, *recA* (G204S) and FRT‐flanked PGK‐gb2 neo cassette	This study
precAD224A1	Suicide plasmid, *recA* (D224S) and FRT‐flanked PGK‐gb2 neo cassette	This study
precAG288Y1	Suicide plasmid, *recA* (G288Y) and FRT‐flanked PGK‐gb2 neo cassette	This study
pFT‐A	Thermonsensitive FLP expression plasmid, Amp^r^	National BioResource Project (NIG, Japan)
pCreA1	Thermonsensitive Cre expression plasmid, Amp^r^	Shimizu et al. (2011)
pTrcHis2A	Expression plasmid, Ampr, *P_trc_*	Invitrogen
pTrcHis2A‐fur	Fur‐His expression plasmid, Ampr, *P_trc_*	This study

Amp^r^, Ampicillin‐resistant; Cm^r^, Chloramphenicol‐resistant.

### 2.2 Reagents and media

NO was generated by four NO donors, namely DETA‐NONOate (DETA/NO) (Cayman Chemical Company, MI, USA), NOC12 (Dojindo Laboratories, Kumamoto, Japan), Spermine‐NONOate (Sper/NO) (Cayman Chemical Company), and PROLI‐NONOate (PROLI/NO) (Cayman Chemical Company). When a heat‐inactivated DETA/NO was prepared, it was dissolved in hydrochloric acid solution (0.1 mol/L) and then incubated for 4 hr at 60°C. The NOS inhibitor *N* ^G^‐Monomethyl‐L‐arginine (L‐NMMA) (Dojindo Laboratries) and an iron‐chelating agent, deferoxamine (Sigma‐Aldrich, MO, USA) were used. NaNO_2_ and NaNO_3_ were purchased from Wako (Tokyo, Japan). LB broth was dissolved in 10 g of Tryptone (Nacalai Tesque, Japan), 5 g of yeast extract (Nacalai Tesque), and 10 g of sodium chloride (Wako) in 1 L of DW, adjusted to pH 7.2 and autoclaved. Polyclonal antisera for Stx1 and Stx2 were prepared as described previously (Noda, Yutsudo, Nakabayashi, Hirayama, & Takeda, 1987; Yutsudo et al., 1987). The anti‐Stx1 and anti‐Stx2 antisera primarily reacted with the A subunit of Stx1 and Stx2, respectively. Anti‐RNA α and anti‐RecA antibodies were obtained from NeoClone Biotechnology International (Madison, WI, USA) and Bio Academia (Osaka, Japan).

### 2.3 Plasmid construction

1 *stx1* promoter plasmids: *stx1* promoter plasmids were constructed by using PCR to amplify a 214‐bp *stx1* promoter DNA fragment from the genomic DNA of EHEC EDL933 using the primer set P1049 ‐ P1050. The DNA fragment was digested with *Nco*I–*Pvu*II (Takara, Tokyo, Japan), and was then cloned into placlux8 (Shimizu, Ohta, Tsutsuki, & Noda, 2011) to yield the plasmid pluxStx1P2. In pluxStx1P2, the *stx1* promoter fragment is placed upstream of the promoter‐less *P. luminescens luxCDABE* gene. To construct the mutated *stx1* promoter plasmid, a PrimeSTAR Mutagenesis kit (Takara) was used with the plasmid DNA of pluxStx1P2 as a template and the primer set P1215 ‐ P1216 to yield the plasmid pluxStx1PGG6 according to the manufacturer's instructions. The construct was confirmed using restriction digestion and DNA sequencing.

2 Template plasmids for homologous recombination: To construct a template plasmid using a Red/ET recombination system for *recA* mutation, the *recA* gene was amplified by PCR from genomic DNA of the EDL933 strain using the primers P1077 and P1078. This fragment was cloned by ligation of a *Not*I fragment into pFRT‐Kan to generate plasmids pFRT‐recA2. Further, to construct template *recA*‐mutated plasmids, a PrimeSTAR Mutagenesis kit (TAKARA) was used with plasmid DNA of pFRT‐recA2 as a template and primer set (Table S1 and S2) to yield template *recA*‐mutated plasmids (Table 1). These constructs were confirmed using restriction digestion and DNA sequencing.

3 Fur expression plasmid: To construct plasmids expressing Fur, *fur* was amplified by PCR from the genomic DNA of the EHEC EDL933 strain using the primer set (P10011 and P715). This DNA fragment was cleaved with *Nco*I and *Sal*I (Takara), and then was inserted into these sites in pTrcHis 2A (Invitrogen, NY, USA) to yield pTrcHis 2A‐fur expressing His‐tagged Fur. The construct was confirmed using restriction digestion and DNA sequencing.

### 2.4 Strain construction

The mutant EHEC strains were derivatives of the EDL933 strain and were obtained by using a Red/ET recombination system (Gene Bridges GmbH, Heidelberg, Germany). Briefly, PCR primers containing 50 bp immediately upstream and downstream, respectively, of the target sequence were used to amplify a DNA fragment containing the FRT‐flanked PGK‐gb2‐neo cassette (Kan^r^) found in appropriate plasmids (Table S3). The linear PCR product was electroporated into the appropriate parent strain which had earlier been transformed with plasmid pRed/ET (amp^r^).

To construct the *stx1* promoter‐deficient EHEC strain EP, *fur*‐deficient EHEC strain EDLf, *Q*‐deficient EHEC strain E1Q1 and *recA*‐deficient EHEC strain ERA1‐1 (Table 1), a DNA fragment containing the Kan^r^ cassette was obtained by PCR from pFRT‐Kan plasmid DNA using the primer sets P613‐P628, P724‐P725, P1064‐P600, and P10002‐P845, respectively. The PCR product was electroporated with the EDL933 previously transformed with plasmid pRed/ET.

To construct *recA* mutants (ERG22Y, ERG24Y, ERE123A, ERG204S, ERD224A and ERG288Y) (Table 1), the respective DNA fragments containing the mutated *recA* gene and Kan^r^ cassette were obtained by PCR from the plasmid DNA of each template *recA*‐mutated plasmid using the primer set P1100 and P845. Each PCR product was electroporated with the ERA1‐1 previously transformed with plasmid pRed/ET. Recombinants containing Kan^r^ in place of the target sequence were selected on Kan plates and confirmed by PCR. Deletion of Kan^r^ elements was completed by transformation with plasmid pFT‐A. Kanamycin‐sensitive mutants were confirmed by PCR. The plasmids pRed/ET and pFT‐A are temperature‐sensitive and were removed by overnight growth at 37°C.

### 2.5 Culture conditions

For anaerobic culture, EHEC strains grown overnight [the optical density at 600 nm (OD_600_) = 1.0 ~ 1.5 × 10^9^ cfu/ml] were diluted 1:100 with LB broth containing 10 mmol/L HEPES (pH 7.0) (LB‐pH 7.0) and grown statically at 37°C using Anaero Pack‐Anaero 5% (Mitsubishi Gas Chemical Company, Tokyo, Japan). For aerobic culture, EHEC strains grown overnight (OD_600_ = 1.0 ~ 1.5 × 10^9^ cfu/ml) were diluted 1:100 with LB‐pH 7.0 and grown statically at 37°C under aerobic conditions.

### 2.6 Cell fractionation

Cells were pelleted by centrifugation at 20,630*g* for 5 min, and the supernatant obtained was used as the culture supernatant fraction. The pellet was suspended in an equal volume of ice‐cold PBS (pH 7.4) and sonicated for 15 s on ice. After sonication, the cell homogenate was centrifuged at 20,630*g* for 5 min, and the supernatant obtained was used as the cell‐associated fraction.

### 2.7 Kinetic analysis of NO release from NO donors under anaerobic conditions

Five microliters of various concentrations of NO donor solution was added to 5 ml of LB‐pH 7.0. To estimate NO release from NO donors, the NO concentration in LB‐pH 7.0 was measured using an amiNO‐2000 NO electrode (Innovative Instruments Inc., Tampa, FL, USA) at 37°C under anaerobic conditions in an anaerobic chamber. The anaerobic chamber (Coy Laboratory Products Inc., Grass Lake, MI, USA) maintains a strict anaerobic (0–5 ppm) environment through a hydrogen gas mix reacting with a palladium catalyst to remove oxygen by forming a water molecule.

### 2.8 NO growth inhibition assay

EHEC strains grown overnight (OD_600_ = 1.0 ~ 1.5 × 10^9^cfu/ml) were diluted 1:100 with LB‐pH 7.0 containing various concentrations of NO donor and grown statically for 18 hr at 37°C under anaerobic conditions. OD_600_ in culture was measured by a spectrophotometer (Ultrospec 3100 pro, GE Healthcare, USA).

### 2.9 Reporter assay

Reporter strains grown overnight (OD_600_ = 1.0 ~ 1.5 × 10^9^ cfu/ml) were diluted 1:100 with LB‐pH 7.0 containing various concentrations of NO donor and grown statically for 18 hr at 37°C under anaerobic conditions. Relative light units (RLU) and the number of bacteria were measured by a GLOMAX 20/20 luminometer (Promega, Madison, USA) and bacteria plate counts (cfu), respectively.

### 2.10 Infection assay

RAW264.7 cells were obtained from the Riken Cell Bank (Tsukuba, Japan) and maintained in Dulbecco's modified Eagle's medium (DMEM; Sigma‐Aldrich) supplemented with 10% fetal bovine serum (FBS) and a 1% antibiotic plus antimycotic solution (Sigma‐Aldrich). The RAW264.7 cells were seeded at 5 × 10^5^ cells per well and then 5 × 10^6^ bacteria of EHEC reporter strains were added to the monolayer per well. The plate was centrifuged briefly to synchronize the infection and then incubated for 20 min (0 hr) at 37°C under an atmosphere of 5% CO_2_. The medium was collected for the measurement of NO_2_^−^ derived from NO under aerobic conditions. The cells were washed and fresh DMEM‐10% FBS containing 100 μg/ml of gentamicin was added to kill the extracellular bacteria. After 2 hr at 37°C under an atmosphere of 5% CO_2_, the medium was collected for measurement of NO_2_^−^, the cells were washed, and the medium was changed to include 12 μg/ml of gentamicin with or without a NOS inhibitor, L‐NMMA (4 mmol/L). The infected monolayers were either lysed from the tissue culture dishes by addition of PBS containing 0.1% deoxycholic acid or further incubated at 37°C under 5% CO_2_. The number of surviving bacteria and RLU were determined by cfu and luminometer, respectively. The concentrations of NO metabolite NO_2_^−^ in medium were determined by Griess assay.

### 2.11 SDS‐PAGE and Immunoblot analysis

Samples were subjected to SDS‐PAGE and electrophoretically transferred to polyvinylidene difluoride (PVDF) membranes (Millipore, USA). After blocking with 5% skim milk in TBS containing 0.1% Tween 20, the membranes were incubated with the indicated antibody, followed by incubation with HRP‐conjugated antirabbit IgG or HRP‐conjugated antimouse IgG (R&D Systems, USA). Antibody‐antigen complexes were detected using an ECL detection kit (SuperSignal^®^ West Pico Chemiluminescent Substrate kit, Thermo Scientific) and an LAS‐1,000 luminescent image analyzer (Fujifilm, Tokyo, Japan). Densitometric analysis was performed by Image Gauge software (Fujifilm), and protein levels were normalized to the α subunit of RNA polymerase (RNA α).

### 2.12 Gb3 receptor ELISA

Porcine erythrocyte Gb3 (Nacalai Tesque) was coated on microtiter plate wells (C96 Maxisorp, Nunc‐immuno plate; Nalge Nunc International, Rochester, NY) by evaporation from an ethanolic solution. A 100‐μl aliquot of ethanolic Gb3 (2 μg/ml for Stx1 and 8 μg/ml for Stx2) was added per microtiter plate well in triplicate, and the ethanol was allowed to evaporate at room temperature for 6 hr. Each well was blocked with 200 μl of 0.2% (w/v) BSA in PBS (BSA‐PBS) for 6 hr and washed twice with 200 μl/well BSA‐PBS. Dilutions of standard Stxs were prepared in BSA‐PBS (Shimizu et al., 2007). The solution was dispensed into the wells and incubated overnight at 4°C. The wells were emptied and washed three times (1 min each) with 200 μl of BSA‐PBS. Then, 100 μl of diluted rabbit antiserum against Stx1 or antiserum against Stx2 in BSA‐PBS was added to the wells for 1 hr at room temperature. The wells were washed as before, then diluted HRP‐conjugate anti‐rabbit IgG in BSA‐PBS was added to the wells and left to sit for 1 hr at room temperature. The substrate, 2,2′‐azino‐bis‐(3‐ethylbenzthiazoline‐6‐sulphonic) diammonium salt (ABTS), was dissolved in 0.1 mol/L citric acid (pH 4.35) at 0.3 mg/ml. A volume of 8.75 μl of 30% H_2_O_2_ was added per 10 ml of solution. The wells were emptied and washed three times with 200 μl of BSA‐PBS for 3 min each. Finally, the wells were washed once with PBS, and 100 μl/well of ABTS solution was added to the plate, which was then shaken gently and placed in the dark. After sufficient color had developed (usually 40–60 min), the absorbance of each well at 415 nm was determined using an ELISA plate reader.

### 2.13 Bacterial mRNA analysis

Total RNA was isolated by ISOGEN II (Wako), and the concentration was determined by measuring the A_260_ value. Subsequently, 0.5 μg of RNA from each sample was reverse‐transcribed by using PrimeScript™ II 1st strand cDNA Synthesis Kit (Takara) according to the manufacturer's protocol. To obtain standard curves for the *hmpA, fur,* and *tufA* genes, genomic DNA from EHEC EDL933 was 10‐fold serially diluted from 1.0 × 10^5^ to 0.1 cfu/2 μl and amplified in the 7,300 Real‐Time PCR System (Applied Biosystems, CA, USA) with the primers (P16 ‐ P17), (P1243 – P1244) or (P890 ‐ P891) using the *Power* SYBER Green PCR Master Mix (Applied Biosystems) according to the manufacturer's protocol, respectively. Serial dilutions of cDNA were amplified in the 7300 Real‐Time PCR System under the same conditions as for the standard curves with the primers (P16 ‐ P17), (P1243 – P1244), or (P890 ‐ P891). The levels of *hmpA, fur,* and *tufA* mRNA were quantified by noting the fluorescence crossing point of the samples on the corresponding standard curve, and the results are presented as the ratio among the expression levels of *hmpA* mRNA, *fur* mRNA, and *tufA* mRNA.

### 2.14 *stx1* promoter assay

Reporter strains grown overnight (OD_600_ = 1.0 ~ 1.5 × 10^9^ cfu/ml) were diluted 1:100 with LB‐pH 7.0 and grown for 2 hr at 37°C, and then 100 μmol/L PROLI/NO or 200 μmol/L deferoxamine was added. Further growth was allowed statically for 20 min at 37°C under anaerobic conditions. RLU and the number of bacteria were measured by a luminometer and bacteria plate counts (cfu).

### 2.15 Statistics

Student's *t*‐test was used to determine significant differences when only two treatment groups were being compared. One‐way ANOVA with Student‐Newman‐Keuls multiple comparisons test was used to analyze significant differences among multiple groups.

## 3 Results

### 3.1 NO inhibits anaerobic growth of the deleted *norV*‐type EHEC

**Figure 1:**
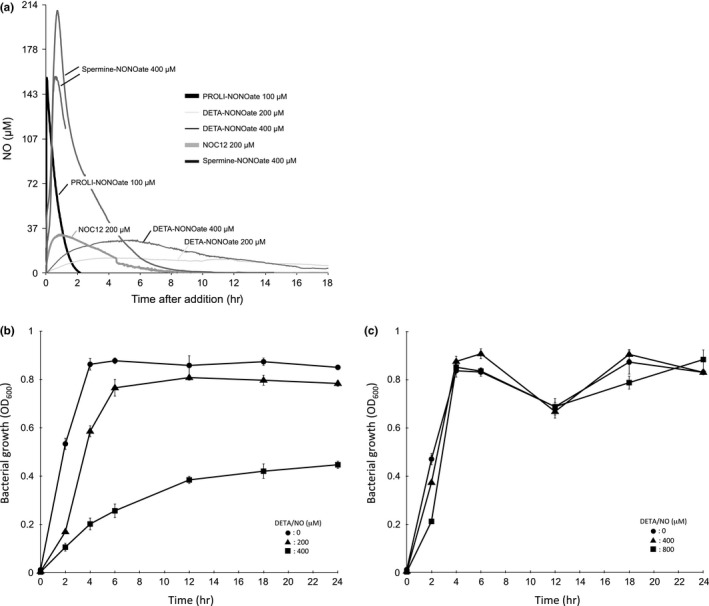
Real‐time quantification of NO concentration and growth of EHEC in the presence of various NO donors under anaerobic conditions. (a) The NO steady‐state levels in LB broth (pH 7.0) in the presence of various NO donors were measured using an amiNO‐2000 NO electrode at 37°C under anaerobic conditions. Representative NO electrode data are shown (*n *=* *3). (b, c) EHEC strains grown overnight were diluted with LB broth containing various concentrations of NO donor and grown statically for 24 hr at 37°C under anaerobic conditions. The OD_600_ in culture was measured by spectrophotometer at the indicated times. Data are the means ± standard deviations of values from three experiments. Results are shown for the deleted *norV*‐type wild EHEC EDL933 (b) and the intact *norV*‐type wild EHEC K15 (c).

The steady‐state concentration of NO depends on both the rate of NO production and rate of NO consumption. NO release from NO donors is solely dictated by temperature and pH level (Thomas et al., 2002), whereas NO consumption depends on the NO reaction with oxygen. To quantitate the precise NO production levels within our assay system, the concentration of NO in LB broth (pH 7.0) treated with different amounts of NO donor was measured using an NO electrode over time at 37°C under anaerobic conditions. The results showed that, under treatment with 200 μmol/L DETA‐NONOate (DETA/NO), the maximum steady‐state concentration of NO in LB medium was 12 μmol/L after 4 hr (Figure 1a). However, the maximum steady‐state concentration of NO was 29 μmol/L after 5 hr of treatment with 400 μmol/L DETA/NO (Figure 1a). Similarly, the maximum steady‐state concentrations of NO in LB medium under the treatments with 100 μmol/L PROLI‐NONOate (PROLI/NO), 200 μmol/L NOC12, and 400 μmol/L Spermine‐NONOate (Sper/NO) were 117 μmol/L after 1.8 min, 25 μmol/L after 1.7 hr, and 140 μmol/L after 0.8 hr, respectively (Figure 1a). Table 2 provides a summary of the peak NO concentration and the time in treatment with various NO donors.

**Table 2:** Peak NO concentration and its time in treatment with various NO donors

	Donor (μmol/L)	Peak NO (μmol/L)	Peak time (hr)
DETA‐NONOate	200	12	4.1
	400	29	4.9
NOC12	200	25	1.7
	400	53	2.2
Spermine‐NONOate	200	29	1.2
	400	140	0.8
PROLI‐NONOate	100	117	0.03

Representative data are shown as the mean (*n *=* *3).

The intact *norV* in EHEC O157 played an important role in protecting the anaerobic growth from NO‐mediated growth inhibition (Shimizu et al., 2012). Therefore, to confirm the growth inhibition of EHEC O157 by NO under anaerobic conditions, the deleted *norV*‐type wild EHEC EDL933 and the intact *norV*‐type wild EHEC K15 were cultured in LB broth with various concentrations of DETA/NO at 37°C. In the deleted *norV*‐type EHEC EDL933, the bacterial growth (OD_600_) was significantly decreased in the presence of 400 μmol/L DETA/NO from 2 hr to 24 hr of treatment compared to that of untreated cells (Figure 1b). When we used 200 μmol/L DETA/NO, the growth of EHEC EDL933 was not inhibited from 12 hr to 24 hr (Figure 1b). In contrast, the bacterial growth of the intact *norV*‐type EHEC K15 was not decreased in the presence of 800 μmol/L DETA/NO from 4 hr to 24 hr under anaerobic conditions (Figure 1c). The deleted *norV*‐type and the intact *norV*‐type EHEC demonstrated different threshold sensitivities of anaerobic growth inhibition to NO.

### 3.2 NO increases Shiga toxin production in EHEC under anaerobic conditions

**Figure 2:**
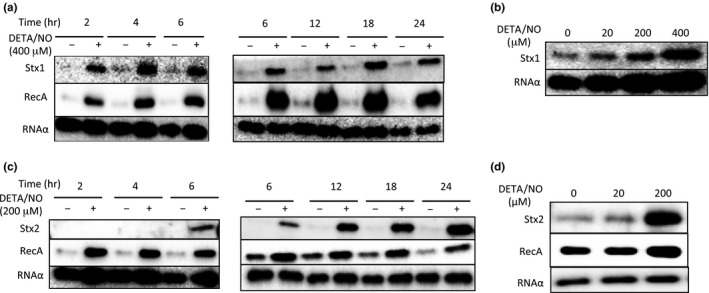
NO enhances Shiga toxin production and RecA expression in the deleted *norV*‐type EHEC under anaerobic conditions. EHEC EDL933 grown overnight was diluted with LB broth containing DETA‐NONOate (DETA/NO) and then grown statically at 37°C under anaerobic conditions. (a) EHEC strains were fractionated into the cell‐associated fractions at the indicated times. A 0.2 μg sample of each protein of the cell‐associated fraction was analyzed by Immunoblot analysis using anti‐Stx1 antiserum, anti‐RecA antibody, and anti‐RNA α antibody as an internal control. (b) EHEC strains were fractionated into the cell‐associated fractions at 18 hr. A 0.2 μg sample of each protein of the cell‐associated fraction was analyzed by Immunoblot analysis using anti‐Stx1 antiserum and anti‐RNA α antibody as an internal control. (c) EHEC strains were fractionated into the cell‐associated fractions and the culture supernatant fractions at the indicated times. A 0.2 μg sample of each protein of the cell‐associated fraction was analyzed by Immunoblot analysis using anti‐RecA antibody and anti‐RNA α antibody as an internal control. Each volume, which corresponds to 0.2 μg of protein of the cell‐associated fraction, of the supernatant fraction was analyzed by Immunoblot analysis using anti‐Stx2 antiserum. (d) EHEC strains were fractionated into the cell‐associated fraction and the culture supernatant fractions at 18 hr. A 0.2 μg sample of each protein of the cell‐associated fraction was analyzed by Immunoblot analysis using anti‐RecA antibody and anti‐RNA α antibody as an internal control. Each volume, which corresponds to 0.2 μg of protein of the cell‐associated fraction, of the supernatant fraction was analyzed by Immunoblot analysis using anti‐Stx2 antiserum.

Next, we investigated the effect of NO on Shiga toxin production in the deleted *norV*‐type EHEC O157 by Immunoblot analysis. Stx1 has been shown to be predominantly located in the cell‐associated fraction, while Stx2 is mainly found in the extracellular fraction (Shimizu et al., 2009). In the presence of 400 μmol/L DETA/NO, Stx1 production in the cell‐associated fraction was increased as compared to that of the control during 2–24 hr incubation (Figure 2a). However, treatment with 200 μmol/L DETA/NO did not enhance Stx1 production (Figure 2b). On the other hand, addition of 200 μmol/L DETA/NO to the bacterial culture enhanced Stx2 production in the culture supernatant from 6 hr to 24 hr (Figure 2c). However, when 20 μmol/L DETA/NO was added, Stx2 productions were not enhanced (Figure 2d). The enhancements of Stx1 and Stx2 production in the deleted *norV*‐type EHEC at the late‐stationary phase were sufficient for exposure to NO at the log phases (Fig.S1). When Gb3‐ELISA was used for quantitative analysis of Stx1 and Stx2, Stx1 production in the cell‐associated fraction of the deleted *norV*‐type EHEC was found to be increased by two–threefold and Stx2 production in the culture supernatant of the deleted *norV*‐type EHEC was increased by ~fivefold as compared to that of the control (Fig. S2). Moreover, transcriptional analysis using C‐P reporter strains revealed that the transcriptional levels of *stx1* and *stx2* in the deleted *norV*‐type EHEC were also enhanced in the treatment with 400 and 200 μmol/L DETA/NO at 18 hr under anaerobic conditions, respectively (Fig. S3). These findings indicated that a high concentration of NO (12–29 μmol/L, maximum steady‐state concentration) is required for enhanced Stx1 production and a low concentration of NO (~12 μmol/L, maximum steady‐state concentration) is sufficient for enhanced Stx2 production in the deleted *norV*‐type EHEC under anaerobic conditions.

The expression of Shiga toxin in EHEC is regulated through induction of the integrated bacteriophage that encodes the Shiga toxin genes. The inductions of Stx‐encoded phages and productions of Shiga toxin are linked to induction of the SOS response, a ubiquitous response to DNA damage (Kimmitt, Harwood, & Barer, 2000). DNA damage activates the bacterial SOS response, leading to activation and upregulation of RecA (Walker, 1984). Thus, we investigated the effect of NO on the expression level of RecA in the deleted *norV*‐type EHEC under anaerobic conditions. When we incubated the deleted *norV*‐type EHEC in a culture containing 200 μmol/L DETA/NO, the expression level of RecA was increased (Figure 2d). However, in the treatment with 20 μmol/L DETA/NO, the amounts of RecA were not enhanced (Figure 2d). Moreover, transcriptional analysis using C‐P reporter strains revealed that the transcriptional levels of *recA* were enhanced in the treatment with 200 and 400 μmol/L DETA/NO at 18 hr under anaerobic conditions (Fig. S3).

**Figure 3:**
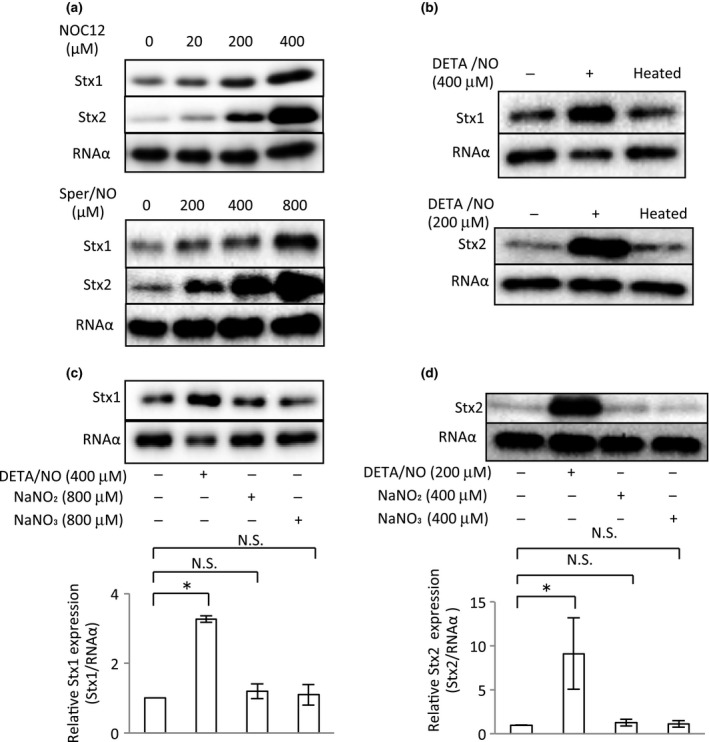
Effect of two kinds of NO donors, inactivated NO donor and NO metabolite, on Shiga toxin production in the deleted *norV*‐type EHEC under anaerobic conditions. EHEC EDL933 grown overnight were diluted with LB broth containing various concentrations of NO donors [NOC12 (a) or Spermine‐NONOate (Sper/NO) (a)], inactivated NO donor (b) or NO metabolite (c, d) and then grown statically for 18 hr at 37°C under anaerobic conditions. The culture supernatant fractions and cell‐associated fractions from the culture of EHEC strains were collected. The cell‐associated fractions were analyzed by Immunoblot analysis using anti‐Stx1 antiserum and anti‐RNA α antibody as an internal control. The culture supernatant fractions were analyzed by Immunoblot analysis using anti‐Stx2 antiserum. The relative amounts of Stx1 and Stx2 were quantified by densitometry and normalized to internal control RNA α . Data are the means ± standard deviations of values from four experiments. *p < 0.01; N. S., not significant.

To further confirm the ability of NO in the deleted *norV*‐type EHEC to enhance Stx1 and Stx2 production, we examined the effects of other NO donors and an inactive NO donor on Shiga toxin production under anaerobic conditions. In the presence of two kinds of NO donors, NOC12 or Spermine‐NONOate (Sper/NO), both Stx1 and Stx2 production in the cell‐associated fractions and the culture supernatant fractions of the deleted *norV*‐type EHEC were increased in a dose‐dependent manner (Figure 3a). Moreover, a heat‐inactivated NO donor did not enhance Stx1 and Stx2 production in the deleted *norV*‐type EHEC (Figure 3b). We considered the possibility that other intermediates of denitrification might induce Shiga toxin production in addition to NO under anaerobic conditions. The nitrate and nitrite of NO metabolites did not enhance Shiga toxin production (Figure 3c, 3d). These results indicated that NO, not intermediates, acts to enhance Stx1 and Stx2 production in the deleted *norV*‐type EHEC under anaerobic conditions.

**Figure 4:**
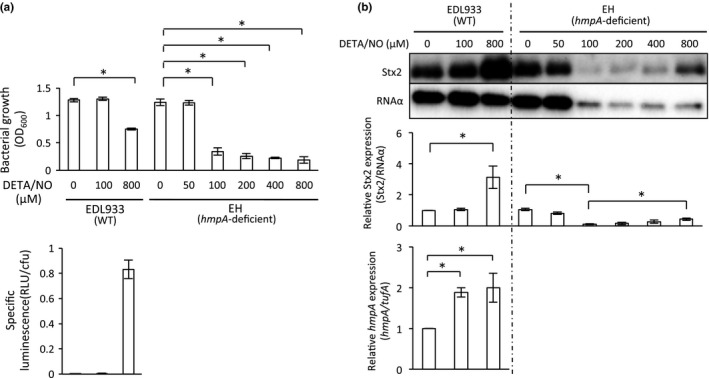
Effect of NO on Stx2 production in wild and *hmpA*‐deficient mutant EHEC strains under aerobic conditions. The deleted *norV*‐type wild EDL933 and the deleted *norV*‐type *hmpA‐*deficient EH grown overnight were diluted with LB broth containing various concentrations of DETA‐NONOate (DETA/NO) and grown statically for 18 hr at 37°C under aerobic conditions. (a) The optical density at 600 nm (OD_600_) was determined. NO level in the deleted *norV*‐type wild EDL933 in medium. EHEC strains harboring the NO reporter plasmid pRPL3 were cultured in LB medium containing a various concentrations of DETA/NO for 18 hr at 37°C under aerobic conditions. Relative light units (RLU) and the number of bacteria were measured by a luminometer and bacteria plate counts (cfu), respectively. Data are the means ± standard deviations of values from three experiments. *p < 0.01. (b) EHEC strains were fractionated into the supernatant fraction and cell‐associated fractions. They were then analyzed by Immunoblot analysis using anti‐Stx2 antiserum and anti‐RNA α antibody, respectively. The relative amounts of Stx2 were quantified by densitometry and normalized to internal control RNA α. The inductions of *hmpA* in wild‐type EHEC were analyzed by real‐time qRT‐PCR. Data are the means ± standard deviations of values from five experiments. *p < 0.01

**Figure 5:**
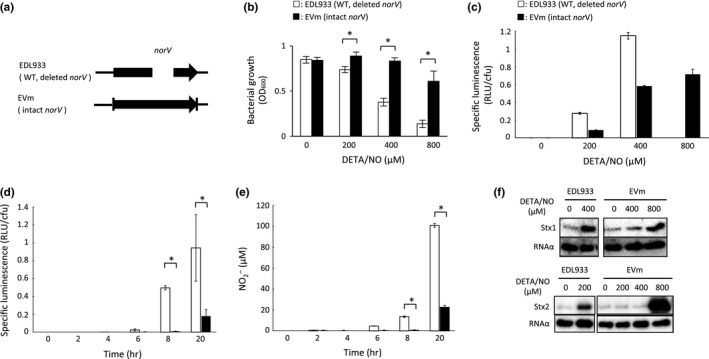
Role of intact NorV in NO‐mediated anaerobic growth inhibition, NO level in bacterial cells and Shiga toxin production in EHEC under anaerobic conditions. (a) Gene structure of the *norV* in the deleted *norV*‐type wild EHEC EDL933 and the intact *norV*‐replacement mutant EHEC EVm. (b) Comparison of NO‐mediated anaerobic growth inhibition between the deleted *norV‐*type EDL933 and the intact *norV*‐type EVm. EHEC strains were cultured in LB medium containing various concentrations of DETA‐NONOate (DETA/NO) for 18 hr at 37°C under anaerobic conditions. The optical density at 600 nm (OD_600_) was measured. Data are the means ± standard deviations of values from five experiments. *p < 0.01. (c) Comparison of the NO level in bacterial cells in medium between the deleted *norV‐*type EDL933 and the intact *norV*‐type EVm. EHEC strains harboring the NO reporter plasmid pRPL3 were cultured in LB medium containing various concentrations (200 or 400 μmol/L for EDL933; 200, 400 or 800 μmol/L for EVm) of DETA/NO for 18 hr at 37°C under anaerobic conditions. Relative light units (RLU) and the number of bacteria were measured by a luminometer and bacteria plate counts (cfu), respectively. Data are the means ± standard deviations of values from three experiments. (d, e) NO level in the deleted *norV‐*type EDL933 within macrophages and the concentration of the NO metabolite NO ^−^_2_ in the culture medium of infected macrophages. The EHEC strain harboring the NO reporter plasmid pRPL3 was added to the monolayer of RAW264.7 cells, and incubated for 20 min (0 hr). The medium was changed to include 100 μg/ml gentamicin. After 2 hr, the cells were washed, and the medium was changed to include 12 μg/ml gentamicin with (black) or without (white) 4 mM NOS inhibitor, L‐NMMA. The infected monolayers were either lysed or further incubated. The RLU and number of surviving bacteria were determined by luminometry and bacteria plate counts (cfu) (d). The concentrations of NO metabolite NO ^−^_2_ in medium were determined by Griess assay (e). Data are the means ± standard deviations of values from three experiments. *p < 0.01. (f) Comparison of NO‐enhanced Shiga toxin production between the deleted *norV‐*type EDL933 and the intact *norV‐*type EVm. EHEC strains were cultured in LB medium containing various concentrations of DETA/NO for 18 hr at 37°C under anaerobic conditions. EHEC strains were fractionated into culture supernatant fractions and cell‐associated fractions. A 0.2 μg sample of each protein of the cell‐associated fraction was analyzed by Immunoblot analysis using anti‐Stx1 antiserum and anti‐RNA α antibody as an internal control. Each volume, which corresponds to 0.2 μg of protein of the cell‐associated fraction, of the supernatant fraction was analyzed by Immunoblot analysis using anti‐Stx2 antiserum.

In previous studies, NO has been shown to inhibit Stx2 production in the deleted *norV*‐type EHEC under aerobic conditions (Vareille et al., 2007). Next, we examined the effect of growth of the deleted *norV*‐type wild EHEC EDL933 in treatment with NO under aerobic conditions. The growth was inhibited by around 40% with 800 μmol/L DETA/NO (Figure 4a), whereas the EHEC growth was inhibited by 50% with 400 μmol/L DETA/NO under anaerobic conditions (Figure 1b). This result suggested that the NO level in bacterial cells treated with 400 μmol/L DETA/NO under anaerobic conditions was higher than that in bacterial cells treated with 800 μmol/L DETA/NO under aerobic conditions. To confirm this, we determined the NO level in bacterial cells treated with an NO donor under aerobic and anaerobic conditions using a novel NO reporter plasmid, pRPL3 (Shimizu et al., 2012). In the deleted *norV*‐type EDL933 (pRPL3), the specific luminescence (RLU/cfu) of the reporter under the treatment with 800 μmol/L DETA/NO after 18 hr was 0.83 ± 0.07 under aerobic conditions (Figure 4a). In contrast, the specific luminescence (RLU/cfu) of the reporter by the treatment with 400 μmol/L DETA/NO was 1.15 ± 0.04 under anaerobic conditions (Figure 5c). These results indicated that the level of NO in the deleted *norV*‐type EHEC EDL933 by treatment with 800 μmol/L DETA/NO under aerobic conditions was lower than that by treatment with 400 μmol/L DETA/NO under anaerobic conditions. Next, we examined the effect of Stx2 production in response to NO under aerobic conditions. In the presence of 800 μmol/L DETA/NO, the production of Stx2 in the deleted *norV*‐type wild EHEC was increased (Figure 4b). When the deleted *norV*‐type wild EHEC was used under aerobic conditions, NO did not inhibit Stx2 production in the presence of 100 μmol/L DETA/NO (Figure 4b). NorV and Hcp in *E. coli* have NO reductase activity under anaerobic conditions (Shimizu et al., 2012; Wang et al., 2016). Under aerobic conditions, HmpA in *E. coli* has been shown to degrade NO to reduce the NO concentration in the aerobic environment (Gardner & Gardner, 2002; Kim et al., 1999). We next analyzed the effect of NO treatment on the aerobic growth and Stx2 production in the deleted *norV*‐type *hmpA*‐deficient mutant EH. The expression levels of *hmpA* mRNA in the deleted *norV*‐type wild EHEC were increased twofold by the treatment with 100 and 800 μmol/L DETA/NO (Figure 4b). Although aerobic growth was inhibited in the treatment with more than 100 μmol/L DETA/NO, Stx2 production was increased in the treatment with 800 μmol/L DETA/NO (Figure 4b). In the treatment with 100 μmol/L DETA/NO, Stx2 production was repressed in the deleted *norV*‐type *hmpA*‐deficient mutant EH (Figure 4b). Therefore, when EHEC were exposed to NO, at lower NO concentrations this exposure promoted the repression of Stx2 production, whereas at higher levels it tended to enhance Stx2 production.

### 3.3 Shiga toxin production in EHEC is influenced by NO reductase in the presence of NO under anaerobic conditions

To clarify the function of NorV in EHEC O157 under anaerobic conditions, we investigated the effect of DETA/NO on anaerobic growth in the deleted *norV*‐type wild EHEC and intact *norV*‐replacement mutant EHEC (Figure 5a). The deleted *norV*‐type wild EDL933 displayed a reduction of anaerobic growth in response to 400 μmol/L DETA/NO (Figure 5b). In contrast, the growth of the intact *norV*‐type EVm was not inhibited by 800 μmol/L DETA/NO (Figure 5b). We next determined the NO level in bacterial cells treated with an NO donor under anaerobic conditions. In the deleted *norV*‐type EDL933 (pRPL3), the specific luminescence (RLU/cfu) of the reporter in the treatments with 200 and 400 μmol/L DETA/NO after 18 hr were 0.28 ± 0.01 and 1.15 ± 0.04, respectively, under anaerobic conditions (Figure 5c). In the intact *norV*‐type EVm (pRPL3), in contrast, the specific luminescence (RLU/cfu) of the reporter in the treatments with 200, 400, and 800 μmol/L DETA/NO were 0.087 ± 0.003, 0.58 ± 0.01, and 0.72 ± 0.06, respectively, under anaerobic conditions (Figure 5c). These results indicated that the deleted *norV*‐type EHEC EDL933 produced a higher level of NO within bacterial cells compared with the intact *norV*‐type EHEC EVm during treatment with the same concentrations of NO donor. We also determined the NO level within macrophages of the deleted *norV*‐type EHEC EDL933 using this NO reporter system. The results showed that both the specific luminescence (RLU/cfu) of the reporter strain within RAW264.7 cells and the concentration of the NO metabolite NO_2_^−^ in the culture medium of infected RAW264.7 cells began to increase after 6 hr postinfection and reached a high level of specific luminescence (0.95 ± 0.37 RLU/cfu) and a high concentration of NO (101 ± 2 μmol/L) at 20 hr postinfection (Figure 5d, 5e). To investigate that the specific luminescence (RLU/cfu) of the reporter strain corresponded to the NO level within macrophages, we used *N* ^G^‐Monomethyl‐L‐arginine (L‐NMMA), which is a specific inhibitor for the NO production of NO‐synthase (NOS) in macrophages. Treatment of 4 mmol/L L‐NMMA did not significantly increase either the specific luminescence (RLU/cfu) of the reporter strain or the concentration of NO_2_^−^ in the culture medium at 20 hr postinfection (Figure 5d, 5e). The specific luminescence (RLU/cfu) of the deleted *norV*‐type NO reporter EHEC EDL933 within RAW264.7 cells at 20 hr postinfection was 0.95 ± 0.37, similar to the level (1.15 ± 0.04) observed with 400 μmol/L DETA/NO after 18 hr of incubation under anaerobic conditions, suggesting that the NO level in the deleted *norV*‐type EHEC EDL933 within RAW264.7 cells was equivalent to that in treatment with 400 μmol/L DETA/NO under anaerobic conditions. These findings suggest that the enhancements of Shiga toxin production in EHEC by NO might occur under physiological conditions, such as within activated macrophages during infection.

Next, to investigate that NO reductase activity suppressed the enhancement of Shiga toxin production by NO under anaerobic conditions, we examined the effect of intact NorV on the NO‐induced Shiga toxin production. The level of production of Stx1 in the deleted *norV*‐type wild EDL933 was increased in the culture using 400 μmol/L DETA/NO (Figure 5f). In the intact *norV*‐type EVm, Stx1 production was not increased in the culture with 400 μmol/L DETA/NO (Figure 5f). In the same way, Stx2 production was also enhanced by 200 μmol/L DETA/NO in the deleted *norV*‐type EHEC EDL933 (Figure 5f). However, NO‐enhanced Stx2 production in the intact *norV*‐type EVm was required for the high concentration of NO donor (800 μmol/L DETA/NO) (Figure 5f).

NO reductase activity of NorV under anaerobic conditions is stronger than that under aerobic conditions. We next examined whether NO reductase activity would affect the NO‐induced enhancement of Shiga toxin production under aerobic conditions. The results showed that the roles played by NO and NO reductase in EHEC for the productions of Stx1 and Stx2 were generally consistent with those under anaerobic conditions, although the effective concentrations of NO donor were much higher than those under aerobic conditions (Fig. S4).

**Figure 6:**
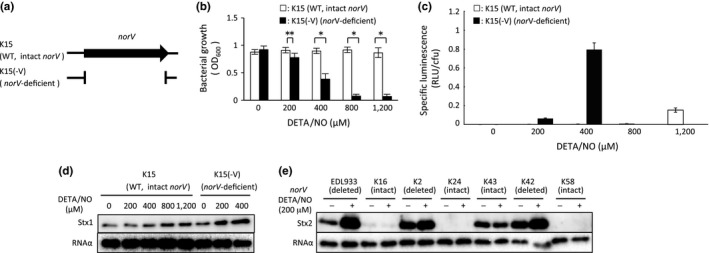
Comparison of NO‐mediated anaerobic growth inhibition, the NO level in bacterial cells and NO‐induced Shiga toxin production between intact *norV‐*type EHEC and *norV‐*deficient EHEC under anaerobic conditions. (a) Gene structure of the *norV* in the intact *norV*‐type wild EHEC K15 and *norV*‐deficient mutant EHEC K15(‐V). (b) Comparison of NO‐mediated anaerobic growth inhibition between the intact *norV‐*type K15 and *norV‐*deficient K15(‐V). EHEC strains were grown with LB medium containing various concentrations of DETA‐NONOate (DETA/NO) for 18 hr at 37°C under anaerobic conditions. The optical density at 600 nm (OD_600_) was monitored. Data are the means ± standard deviations of values from five experiments. *p < 0.01; **p < 0.05. (c) Comparison of the NO level in bacterial cells in medium between the intact *norV‐*type K15 and the *norV‐*deficient K15(‐V). EHEC strains harboring the NO reporter plasmid pRPL3 were cultured in LB medium containing various concentrations [200, 400, 800, or 1200 μmol/L for K15; 200 or 400 μmol/L for K15(‐V)] of DETA/NO for 18 hr at 37°C under anaerobic conditions. Relative light units (RLU) and the number of bacteria were measured by a luminometer and bacteria plate counts (cfu), respectively. (d) Comparison of NO‐enhanced Shiga toxin production between the intact *norV*‐type K15 and *norV*‐deficient K15(‐V). EHEC strains grown containing various concentrations of DETA/NO for 18 hr at 37°C under anaerobic conditions. EHEC strains were fractionated into culture supernatant fractions and cell‐associated fractions. A 0.2 μg sample of each protein of the cell‐associated fraction was analyzed by Immunoblot analysis using anti‐Stx1 antiserum and anti‐RNA α antibody as an internal control. (e) *E. coli* strains were cultured with LB broth containing 200 μmol/L DETA/NO for 18 hr at 37°C under anaerobic conditions. EHEC strains were fractionated into culture supernatant fractions and cell‐associated fractions. Each volume, which corresponds to 0.2 μg of protein of the cell‐associated fraction, of the supernatant fraction was analyzed by Immunoblot analysis using anti‐Stx2 antiserum. A 0.2 μg sample of each protein of the cell‐associated fraction was analyzed by Immunoblot analysis using anti‐RNA α antibody as an internal control.

In this study, EDL933, a deleted *norV*‐type strain of EHEC, was used as the parent EHEC, and also we used the intact *norV*‐type wild EHEC K15 as a parent strain (Figure 6a). We next investigated the effect of NO reductase in EHEC on Stx1 and Stx2 productions under anaerobic conditions using wild type K15 and the norV‐deficient K15(‐V) strains. Growth in the intact *norV*‐type EHEC K15 was not inhibited even in the presence of 1200 μmol/L DETA/NO (Figure 6b). However, the *norV*‐deficient K15(‐V) dramatically decreased anaerobic growth in response to 400 μmol/L DETA/NO (Figure 6b). The intact *norV*‐type EHEC K15 produced a lower level of NO within bacterial cells compared with the *norV*‐deficient K15(‐V) following treatment with the same concentrations of DETA/NO (200 or 400 μmol/L) (Figure 6c).

Stx1 production in intact *norV*‐type EHEC K15 was only increased in the presence of 1200 μmol/L DETA/NO (Figure 6d). In the *norV*‐deficient K15(‐V), Stx1 production was increased by culture with 400 μmol/L DETA/NO (Figure 6d). Next, to confirm that the enhancement of Stx2 production in EHEC by the addition of an NO donor was suppressed by intact NorV under anaerobic conditions, we examined the enhancement of Stx2 production in four additional Stx2‐producing EHEC strains by Immunoblot analysis. When EHEC were incubated with 200 μmol/L DETA/NO, the deleted *norV*–type wild EHEC strains (K2, K42) exhibited the enhancement of Stx2 production (Figure 6e). The intact *norV*‐type wild EHEC strains (K16, K43) did not show enhanced Stx2 production in the culture containing 200 μmol/L DETA/NO (Figure 6e).

### 3.4 Mechanism of the enhancement of Stx1 production by NO under anaerobic conditions

**Figure 7:**
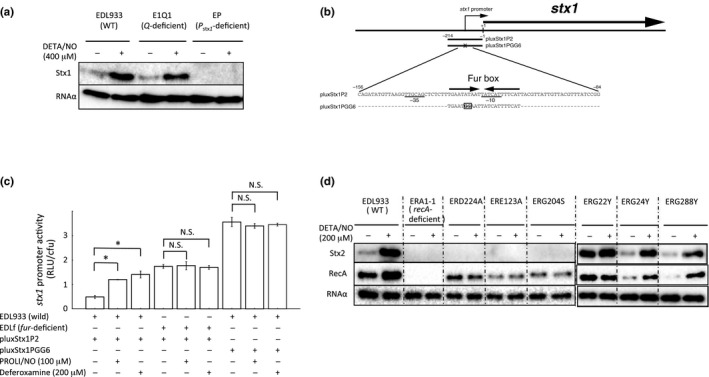
Effects of NO on Stx1 and Stx2 production in various mutant EHEC under anaerobic conditions. (a) Effects of NO on Stx1 production in wild EHEC EDL933, *Q*‐deficient mutant EHEC E1Q1 and *P*_stx1_‐deficient mutant EHEC EP under anaerobic conditions. EHEC were cultured in medium containing 400 μmol/L DETA‐NONOate (DETA/NO) for 18 hr at 37°C under anaerobic conditions. EHEC were fractionated into cell‐associated fractions. A 0.2 μg sample of each protein of the cell‐associated fraction was analyzed by Immunoblot analysis using anti‐Stx1 antiserum and anti‐RNA α antibody as an internal control. (b) Promoter sequence of *stx1* and schematic representation of various *stx1* promoter‐*luxCDABE* fusion genes utilized in the mutation analysis. The arrows indicate a Fur box, and the boxed region within the Fur box is a mutated site. The −35 and −10 regions of the proposed promoter are underlined. The number indicates the nucleotide position with base pairs upstream of the start codon (+1) of *stx1*. (c) Comparison of the specific luminescence of EDL933 harboring an *stx1* reporter plasmid, pluxStx1P2, *fur*‐deficient EDLf harboring pluxStx1P2 and EDL933 harboring a mutated *stx1* reporter plasmid, pluxStx1PGG6. Reporters were cultured in LB broth supplemented with or without 100 μmol/L PROLI‐NONOate (PROLI/NO) or 200 μmol/L deferoxamine at 37°C and then were collected after 20 min for the estimation of specific luminescence. Data are the means ± standard deviations of values from three experiments. *p < 0.01; N.S., not significant. (d) Effects of NO on Stx2 production in the EHEC EDL933, *recA*‐deficient mutant EHEC ERA1‐1 and *recA* point‐mutant EHEC strains under anaerobic conditions. EHEC strains were grown with LB broth containing 200 μmol/L DETA‐NONOate (DETA/NO) for 18 hr at 37°C under anaerobic conditions. EHEC strains were fractionated into culture supernatant fractions and cell‐associated fractions. Each volume, which corresponds to 0.2 μg of protein of the cell‐associated fraction, of the supernatant fraction was analyzed by Immunoblot analysis using anti‐Stx2 antiserum. The cell‐associated fraction protein was analyzed by Immunoblot analysis using anti‐RecA antibody and anti‐RNA α antibody as an internal control.

With respect to the Stx1 expression in EHEC, there are two types of promoters in the Stx1‐encoding phage of EHEC (Shimizu et al., 2009). The first is a *P*_Stx1_, which is adjacent to the *stx1* (not the *stx2*) and regulated by the environmental iron concentration in EHEC (Calderwood & Mekalanos, 1987). A functional Fur consensus box exists in the *P*_Stx1_ region (Calderwood & Mekalanos, 1987, 1988). Fur is a global repressor that is regulated by iron (Hantke, 2001). The second type of promoter is transcribed from the *P*_R_' of the Stx1‐encoding phage, which is important for Stx1 production (Wagner et al., 2002). Therefore, to examine whether the *P*_Stx1_ or the *P*_R_' of the Stx1‐encoding phage regulates NO‐induced Stx1 production under anaerobic conditions, we constructed *Q* of the Stx1‐encoding phage‐deficient mutant E1Q1 and the *P*_Stx1_‐deficient mutant EP. Since *Q* of the Stx1‐encoding phage is a late antiterminator gene (Wagner & Waldor, 2002; Wagner et al., 2002), induction of the Stx1‐encoding phage in E1Q1 could not occur. In the *Q*‐deficient E1Q1, the expression of Stx1 was increased by the NO‐donor treatment to the same degree as in the wild‐type strain (Figure 7a). In the *P*_Stx1_‐deficient mutant, however, the level of Stx1 production was much lower than that in the wild‐type EHEC (Figure 7a). These results indicated that the enhancement of Stx1 production was related with the *P*_Stx1_, which was regulated by the iron regulator Fur. To determine whether NO treatment affected the *fur* expression in EHEC, we analyzed the expression levels of *fur* mRNA. The expression level of *fur* in EHEC at log phase in treatment with 400 μmol/L DETA/NO was equivalent to that of the control under anaerobic conditions (Figure S5). Next, to investigate that derepression of Fur contributes to the enhancement of Stx1 production in EHEC treated with an NO donor, we constructed the *stx1* reporter plasmid pluxStx1P2 (Figure 7b). The *P*_Stx1_ activity (RLU/cfu) of EDL933 harboring the *stx1* reporter plasmid pluxStx1P2, EDL933 (pluxStx1P2), was increased in treatment with either 100 μmol/L PROLI‐NONOate (PROLI/NO) or 200 μmol/L of an iron chelator, deferoxamine (Figure 7c). The *P*_Stx1_ activity (RLU/cfu) of the *fur*‐deficient mutant EDLf (pluxStx1P2) was increased at the basal level. But, it was not enhanced following treatment with either 100 μmol/L PROLI/NO or 200 μmol/L deferoxamine alone (Figure 7c). When we used the Fur box‐mutated reporter plasmid pluxStx1PGG6, the level of the *P*_Stx1_ activity (RLU/cfu) of EDL933 (pluxStx1PGG6) was similar to those of EDL933 (pluxStx1PGG6) in treatment with 100 μmol/L PROLI/NO or 200 μmol/L deferoxamine, respectively (Figure 7b, c). These results indicated that the inhibition of Fur binding to the Fur box in the *P*_Stx1_ region functions to enhance the *P*_Stx1_ activity in response to NO.

### 3.5 Mechanism of the enhancement of Stx2 production by NO under anaerobic conditions

Because, in the Stx2‐encoding phage, there was no promoter for Stx2 production adjacent to *stx2*, the *recA*‐deficient mutant EHEC ERA1‐1 did not produce Stx2 (Figure 7d). Therefore, we could not investigate the effect of NO on RecA‐dependent Stx2 expression using the *recA*‐deficient mutant. A previous study (Adikesavan et al., 2011) reported that the introduction of a point mutation of *recA* (e.g., E123A, G204S, or D224A) leads to the loss of upregulation of RecA in each *recA*‐deficient *E. coli* strain in the presence of nalidixic acid (100 μg/ml), while the point mutations G22Y, G24Y, and G288Y of *recA* do not impair the upregulation of RecA (Table S4). The mutations of G24Y and G288Y of *recA* continued to cleave LexA in the presence of nalidixic acid (100 μg/ml), but that of G22Y of *recA* lead to upregulation of RecA without LexA cleavage upon DNA damage (Table S4) (Adikesavan et al., 2011). These results suggested that one of the functions of RecA in *E. coli* allows *E. coli* to separate the activation of RecA and induction of SOS response. To investigate whether RecA affects the enhancement of Stx2 production in the presence of an NO donor under anaerobic conditions, we constructed the same isogenic *recA* point‐mutated strains using EHEC EDL933 as a parent strain (Table 1 and Table S4). Wild or *recA* point‐mutated EHEC strains (e.g., ERG123A, ERG204S, ERD224A) were cultured with LB broth containing 200 μmol/L DETA/NO for 18 hr under anaerobic conditions (Figure 7d). Stx2 production was not detected in the ERE123A, ERG204S, or ERD224A strains in the presence or absence of an NO donor, which was similar to the findings for the *recA*‐deficient ERA1‐1. The levels of RecA were increased by exposure to NO after 18 hr of incubation in the wild‐type, ERG24Y and ERG288Y strains. In the *recA*‐mutant ERG22Y strain, the level of RecA was similar to that of the wild‐type strain in the absence of NO‐donor treatment, and RecA was not increased by addition of the NO donor. Moreover, in *recA*‐mutant ERG22Y, the basal levels of Stx2 production were increased in comparison with those of the wild‐type strain. However, the NO‐enhanced Stx2 production was not observed in *recA*‐mutant ERG22Y. On the other hand, in the *recA*‐mutant strains, ERG24Y and ERG288Y, Stx2 production in the culture with an NO donor was enhanced to a degree similar to that in the wild‐type strain. These results indicated that the increase of RecA in EHEC was related to the NO‐enhanced Stx2 production under anaerobic conditions.

## 4 Discussion

The steady‐state NO concentration has emerged as a key determinant of bacterial response. Our data have revealed the approximate threshold concentration limits for growth inhibition, activation of Stx1 production and activation of Stx2 production in EHEC under anaerobic conditions. The results indicated that there were three concentration levels of NO activity in the deleted *norV*‐type EHEC EDL933 under anaerobic conditions: a growth inhibition range (12–29 μmol/L, maximum steady‐state concentration), an enhancement of Stx1 production range (12–29 μmol/L, maximum steady‐state concentration), and an enhancement of Stx2 production range (~12 μmol/L, maximum steady‐state concentration). This suggested that the bacterial responses were differentially influenced by specific NO concentrations. In this study, we used four kinds of NO donors (DETA/NO, PROLI/NO, NOC12, and Sper/NO) as the source of NO. Under anaerobic conditions, our results were consistent with previous reports under aerobic conditions (Thomas et al., 2004, 2008), namely, the steady‐state concentrations of NO in medium were much lower than those of the NO donors, except in the case of PROLI/NO.

Macrophages are an important component of the innate immune response. One of the antimicrobial systems of macrophages is the iNOS pathway, which is responsible for the generation of NO (Bogdan, 2001; MacMicking, Xie, & Nathan, 1997; Nathan & Hibbs, 1991). We observed that the specific luminescences (RLU/cfu) of the deleted *norV*‐type NO reporter within macrophages were 0.50 ± 0.02, which corresponds to the enhancement of Stx2 production, and 0.95 ± 0.37, which corresponds to the enhancement of Stx1 production, at 8 hr and 20 hr postinfection, respectively (Figure 5d). These results suggested that the NO generated in macrophages might induce Stx2 production in EHEC after 8 hr postinfection, and enhancement of Stx1 production in EHEC by NO within macrophages might occur at 20 hr postinfection or later. However, it was previously reported that NO suppressed the activation of RecA and then inhibited Stx2 synthesis in EHEC (Vareille et al., 2007). These results appear to be similar to our results under aerobic conditions using an *hmpA*‐deficient mutant strain (Figure 4b). The transcriptional repressor NsrR has a low threshold for sensing NO relative to its lesser sensitivity for SOS response (Karlinsey et al., 2012; Spek et al., 2001). The inhibition of *stx2* expression in EHEC was essential for a long‐term exposure to a significant level of NO (Vareille et al., 2007). Under our experimental conditions, Stx2 production alone might have been inhibited by treatment with 100 μmol/L DETA/NO at 18 hr of incubation under aerobic conditions when we used the deleted *norV*‐type *hmpA*‐deficient EHEC. At lower concentrations of NO, RecA activation and Stx2 production might be inhibited by NO via the NsrR (Vareille et al., 2007). On the other hand, at higher concentrations of NO, RecA in EHEC might be activated by NO to induce an SOS response.

As an iron‐containing transcriptional regulator, Fur has been shown to directly respond to NO in *E. coli* (Fleischhacker & Kiley, 2011; Mukhopadhyay, Zheng, Bedzyk, LaRossa, & Storz, 2004; Spiro, 2006). As a transcriptional repressor, Fur regulates Stx1 production via *P*_Stx1_ in EHEC and is inactivated by exposure to NO, and then the derepression of Fur‐regulated *P*_Stx1_ is observed in EHEC. In contrast, the induction of the SOS response is caused by NO, resulting in the indirect activation of RecA (Lobysheva et al., 1999; Schapiro et al., 2003). We revealed that a higher concentration of NO is required for enhanced Stx1 production than for enhanced Stx2 production in EHEC. These results indicated that the concentration of NO required to depress Fur was higher than that required to activate RecA in EHEC, suggesting that Fur has a high threshold for sensing NO (Karlinsey et al., 2012). When Fur was overexpressed in EHEC, there was no significant inhibition of the enhancement of Stx1 production by NO‐donor treatment (Fig. S6). Fur is also a repressor of *hmpA* transcription in *E. coli* (Hernandez‐Urzua et al., 2007). Thus, not only inhibition of NO‐enhanced Stx1 production but also repression of *hmpA* expression might occur through the overexpression of Fur in EHEC.

It was reported that an *recA*‐deficient *E. coli* strain harboring a mutant *recA* expression plasmid (G288Y, G22Y, or G24Y) exhibited upregulation of RecA levels after DNA damage, while an *recA*‐deficient *E. coli* strain harboring a mutant *recA* expression plasmid (E123A, D224A, or G204S) did not (Adikesavan et al., 2011) (Table S4). Our results suggest that, in order for Stx2 to be expressed in EHEC, higher level expression of RecA or upregulation of RecA in response to DNA damage might be necessary. Moreover, *E. coli* (*recA* G24Y) exhibited both upregulation of RecA and LexA cleavage activity after DNA damage, but *E. coli* (*recA* G22Y) exhibited upregulation of RecA even in the absence of LexA cleavage activity (Adikesavan et al., 2011) (Table S4). Both *E. coli* (*recA* G24Y) and *E. coli* (*recA* G22Y) showed the UmuD cleavage activity (Adikesavan et al., 2011) (Table S4). Although the interaction of LexA with activated RecA triggers the cleavage reaction, activated RecA can also mediate the cleavage of two other groups of proteins. The first is a group of temperate phage repressors, exemplified by the λ CI repressor, which is cleaved in lysogens upon SOS‐induction treatment. The second set of substrates is a set of mutagenesis proteins, exemplified by the host UmuD protein, which is activated by specific cleavage to perform specific roles in SOS mutagenesis. Since the ERG22Y mutant strain did not exhibit an enhancement of Stx2 production when cultured in the presence of an NO donor, the LexA cleavage activity, not UmuD cleavage activity, of activated RecA in EHEC was related to NO‐enhanced Stx2 production, and the λ CI repressor of Stx2‐encoding phage in EHEC might be cleaved by the LexA cleavage activity of RecA to enhance Stx2 production (Table S4).

## Conflict of Interest

None declared.
