# Supplementary material for: Nitric oxide‐enhanced Shiga toxin production was regulated by Fur and RecA in enterohemorrhagic Escherichia coli O157
Source: Microbiologyopen. 2017 Mar 15;6(4):e00461. doi: 10.1002/mbo3.461 (PMC5552940; doi:10.1002/mbo3.461)
Supplement: Supplementary file 1 [file MBO3-6-na-s001.pdf]

Fig. S1

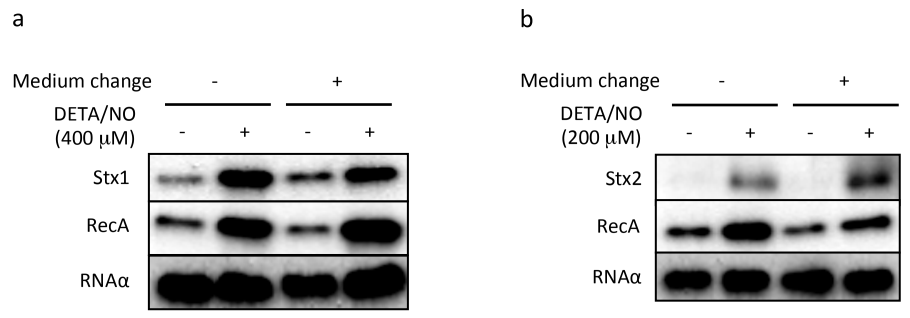

Supplemental Figure 1
The enhancements of Shiga toxin production and RecA expression in the deleted *norV*-type EHEC are sufficient for exposure to NO at the log phase.
EHEC EDL933 grown overnight was diluted with LB broth containing DETA-NONOate (DETA/NO) and then grown statically at 37°C for 18 h under anaerobic conditions as a control. After 6 h the cells were washed, and medium was changed to LB broth without an NO donor. EHEC were further incubated for 12 h at 37°C under anaerobic conditions.
(a) EHEC strains were fractionated into cell-associated fractions. A 0.2 μg sample of each protein of the cell-associated fraction was analyzed by immunoblot analysis using anti-Stx1 antiserum, anti-RecA antibody and anti-RNA α antibody as an internal control.
(b) EHEC strains were fractionated into the cell-associated fractions and the culture supernatant fractions. A 0.2 μg sample of each protein of the cell-associated fraction was analyzed by immunoblot analysis using anti-RecA antibody and anti-RNA α antibody as an internal control. Each volume, which corresponds to 0.2 μg of protein of the cell-associated fraction, of the supernatant fraction was analyzed by immunoblot analysis using anti-Stx2 antiserum.

Fig. S2

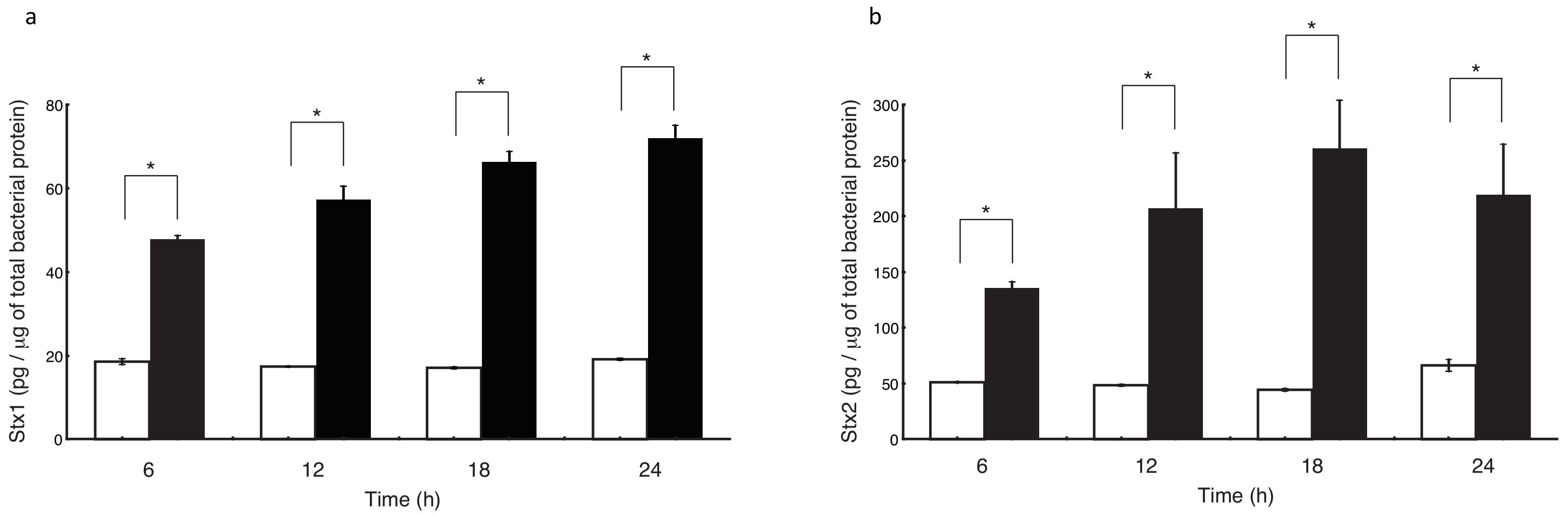

Supplemental Figure 2
NO enhances the production of Shiga toxins in the deleted *norV*-type EHEC under anaerobic conditions.
EHEC EDL933 were cultured in LB medium containing various concentrations of DETA-NONOate (DETA/NO) at 37°C under anaerobic conditions. EHEC strains were fractionated into cell-associated fractions and supernatant fractions at the indicated times. The amount of Stx1 in the cell-associated fraction (a) and the amount of Stx2 in the supernatant fraction (b) were measured by Gb3-ELISA. The amounts of total proteins in the cell-associated fraction were determined by Bradford method.
Data are the means ± standard deviations of values from three experiments. *$p < 0.01$.

Fig. S3

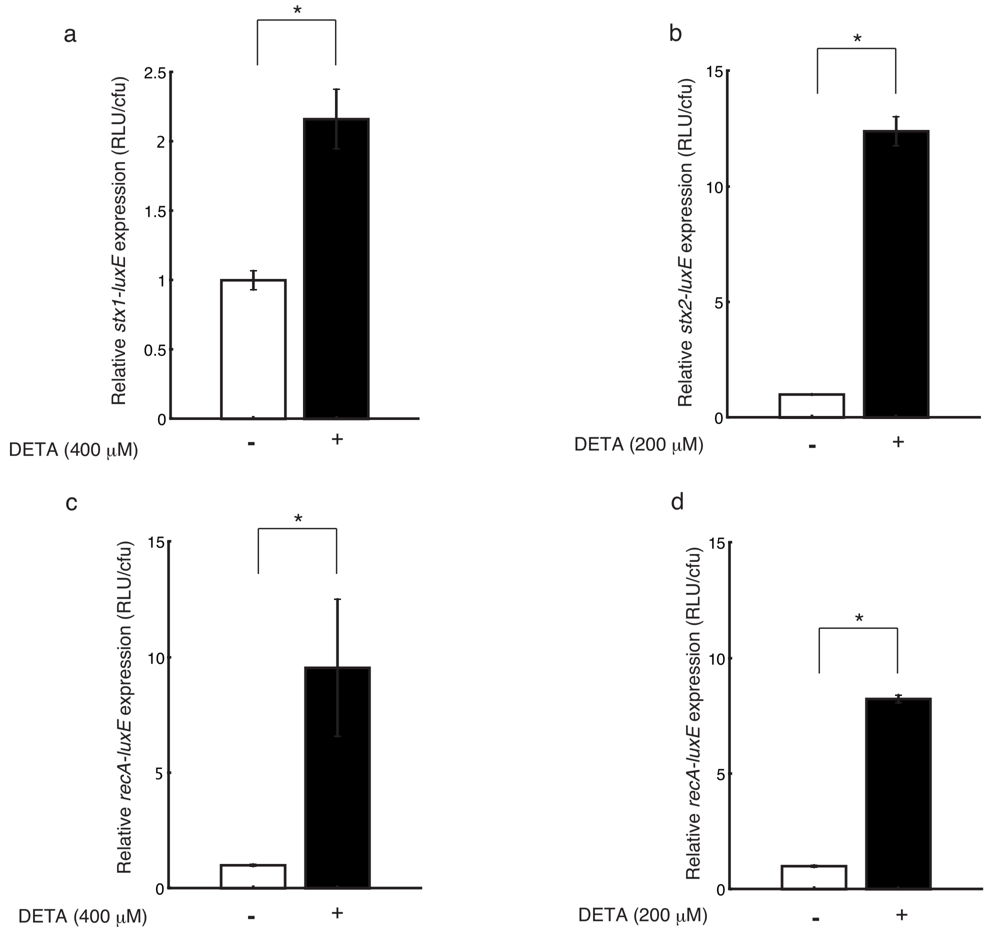

Supplemental Figure 3

NO enhances transcriptions of *stx1*, *stx2* and *recA* in the deleted *norV*-type EHEC under anaerobic conditions.

EHEC reporter strains were cultured in LB medium containing various concentrations of DETA-NONOate (DETA/NO) for 18 h at 37°C under anaerobic conditions.

Relative light units (RLU) and the number of bacteria were measured by a luminometer and bacteria plate counts (cfu), respectively.

(a) *stx1*-reporter E1-E2S (pluxCDAB3) treated with or without 400 μM DETA/NO.

(b) *stx2*-reporter E(SR)2-E1S (pluxCDAB3) treated with or without 200 μM DETA/NO.

(c) *recA*-reporter ERSA (pluxCDAB3) treated with or without 400 μM DETA/NO.

(d) *recA*-reporter ERSA (pluxCDAB3) treated with or without 200 μM DETA/NO.

Data are the means ± standard deviations of values from three experiments. $^*p < 0.01$.

Fig. S4

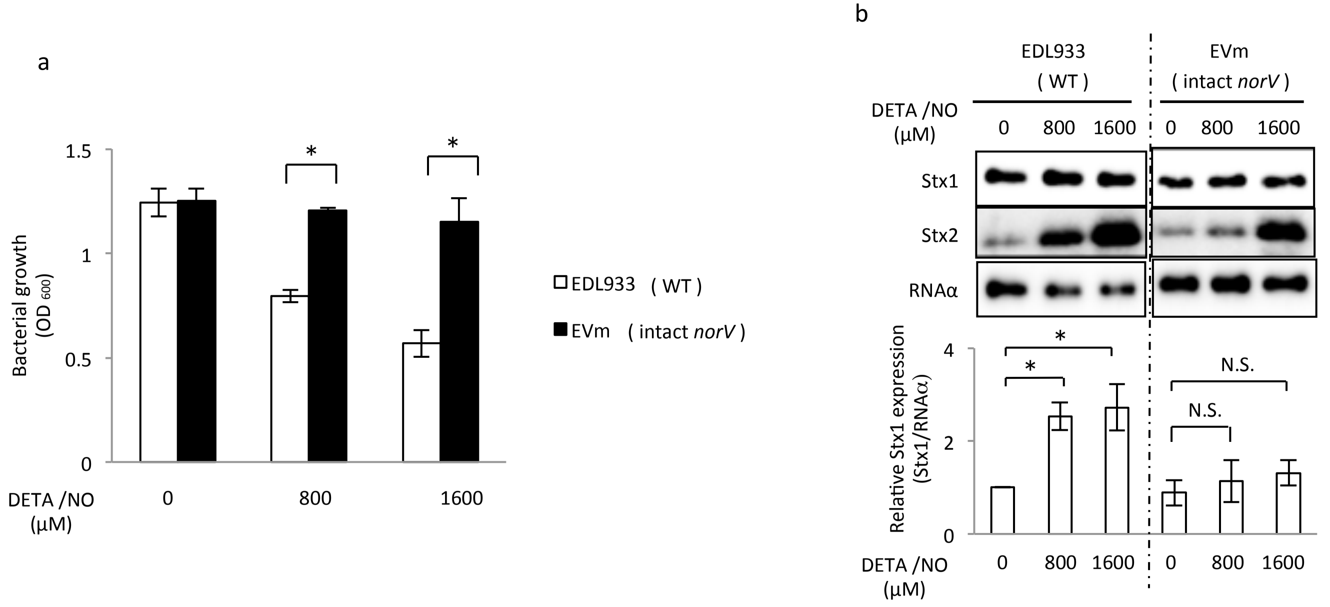

Supplemental Figure 4

Role of intact NorV in NO-mediated aerobic growth inhibition and Shiga toxin production in EHEC under aerobic conditions.

EHEC strains grown overnight were diluted with LB medium containing various concentrations of DETA-NONOate (DETA/NO) and grown statically for 18 h at 37°C under aerobic conditions.

(a) The optical density at 600 nm (OD_{600}) was determined. Data are the means ± standard deviations of values from four experiments. $*p < 0.01$.

(b) EHEC strains were fractionated into culture supernatant fractions and cell-associated fractions. A 0.2 μg sample of each protein of the cell-associated fraction was analyzed by immunoblot analysis using anti-Stx1 antiserum and anti-RNA α antibody as an internal control. Each volume, which corresponds to 0.2 μg of protein of the cell-associated fraction, of the supernatant fraction was analyzed by immunoblot analysis using anti-Stx2 antiserum. The relative amounts of Stx1 and Stx2 were quantified by densitometry and normalized to internal control RNA α. Data are the means ± standard deviations of values from four experiments. $*p < 0.01$; N.S. , not significant.

Fig. S5

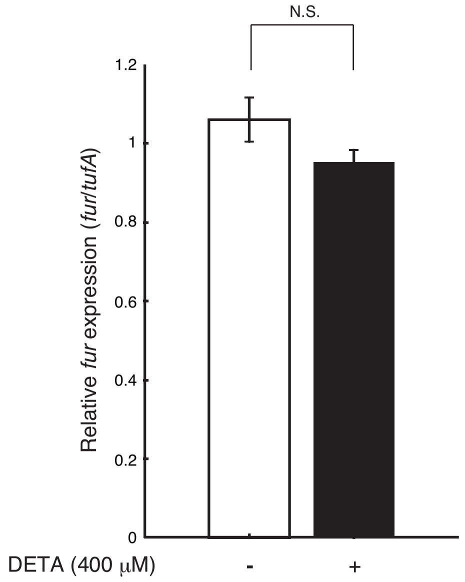

Supplemental Figure 5
Effect of NO on *fur* expression in the deleted *norV*-type EHEC under anaerobic conditions. EHEC strains grown overnight were diluted with LB medium containing 400 μM DETA-NONOate (DETA/NO) and grown statically for 6 h at 37°C under aerobic conditions. The expression level of *fur* mRNA in the deleted *norV*-type EHEC was analyzed by real-time RT-PCR. Data are the means ± standard deviations of values from three experiments. N.S. , not significant.

Fig. S6

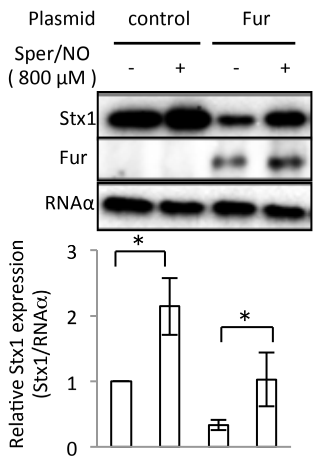

Supplemental Figure 6
Effect of Fur overexpression on NO-enhanced Stx1 production in EHEC under anaerobic conditions. EHEC EDL933 harboring the Fur expression plasmid (pTrcHis2A-fur) or a control plasmid (pTrcHis2A) were cultured in LB medium containing 800 μM NO donor Spermine NONOate (Sper/NO) with or without 1 mM IPTG for 18 h at 37°C under anaerobic conditions. EHEC strains were fractionated into cell-associated fractions. The samples were analyzed by immunoblot analysis using anti-Stx1 antiserum, anti-His Tag antibody for detection of Fur-His and anti-RNA α antibody as an internal control. The relative amounts of Stx1 were quantified by densitometry and normalized to internal control RNA a. Data are the means ± standard deviations of values from four experiments. *$p < 0.01$.

Supplemental Table S1 PCR primers

Primer	Sequence	Reference
P16	GTGGCTGGGAAGGTACTCG	Svensson *et al.* (2010)
P17	AGCCAGACGCCGAGATATTG	Svensson *et al.* (2010)
P600	GACCGAGGTAGCCAGAATACTGAACTGTAGTCGCAACACGGTCAGAAAATTAACCCTCACTAAAGGGCG	This study
P613	TCTACGAGTTTGCCAGCCTCCCCCAGTGGCTGGCTTTTTTATGTCCGTAATTAACCCTCACTAAAGGGCG	Shimizu *et al.* (2009)
P628	CCACCCCCTGAAGGACGGCGTTTTACGGCGCACCGGATAAACGTAACTAATACGACTCACTATAGGGCTC	Shimizu *et al.* (2009)
P715	GGCCATGGCCGCTTAGTAACAGGAC	This study
P724	ATTAACAATATTTGCCAGGGACTTGTGGTTTTCATTTAGGCGTGGCAATTAACCCTCACTAAAGGGCG	This study
P725	ACAAATAAGTGAGAGCTGTAACTCTCGCTTTTCTTATTTCCCTTGCATAATACGACTCACTATAGGGCTC	This study
P845	TACAACTTAAAAAGCAAAAGGGCCGCAGATGCGACCCTTGTGTATCAAATACGACTCACTATAGGGCTC	This study
P846	TCGCTGCGCCGTTTAAA	This study
P890	TGGTTGATGACGAAGAGCTG	Vareille *et al.* (2007)
P891	GCTCTGGTTCCGGAATGTAA	Vareille *et al.* (2007)
P1049	CCCAGCTGCCTGTGTATCAATAAATGTT	This study
P1050	GGCCATGGATTACACAATACTCCTTGAG	This study
P1064	GGGTTAAGCAGCGTGGCAATGTAACCACTCTTATCATGATATGCAGAAATACGACTCACTATAGGGCTC	This study
P1077	GGGCGGCCGCGATCAGGTCGCGGTAAT	This study
P1078	GGGCGGCCGCAAGACGATTAAAAATCTTCGTT	This study
P1079	CAATTTTATAAAGGCTCCATCATG	This study
P1080	GCCTTTATAAAATTGTTTCTCAAT	This study
P1081	GGTAAATACTCCATCATGCGCCTG	This study
P1082	GATGGAGTATTTACCAAATTGTTT	This study
P1085	ACCGGCGCGCAGGCACTGGAAATC	This study
P1086	TGCCTGCGCGCCGGTGTCCGGCTG	This study
P1089	ATGTTCAGTAACCCGGAAACCACT	This study
P1090	CGGGTTACTGAACATCACACCAAT	This study
P1093	CGTCTCGCCATCCGTCGTATCGGC	This study
P1094	ACGGATGGCGAGACGAACAGAGGC	This study
P1095	AAAGCATACGCGTGGTACAGCTAC	This study
P1096	CCACGCGTATGCTTTCTCGATCAG	This study
P1100	GCGATCAGGTCGCGGTAAT	This study
P1215	TTTGAATGGAATTATCATTTTCATTAC	This study
P1216	ATAATTCCATTCAAAGAGAGCTGCAA	This study
P1243	GTATCGTCACCCGCCACAA	Yang *et al.* (2015)
P1244	CGCAGTGACCGTAAAGATAGAGA	Yang *et al.* (2015)
P10011	GGGTCGACTTTGCCTTCGTGCGCGT	This study
P10002	AACAGAACATATTGACTATCCGGTATTACCCGGCATGACAGGAGTAAAATTAACCCTCACTAAAGGGCG	This study

References

Svensson, L., Poljakovic, M., Save, S., Gilberthorpe, N., Schon, T., Strid, S., … Persson, K. (2010). Role of flavohemoglobin in combating nitrosative stress in uropathogenic *Escherichia coli*--implications for urinary tract infection. Microb pathog 49: 59-66.

Yang, B., Feng, L., Wang, F. and Wang, L. (2015). Enterohemorrhagic *Escherichia coli* senses low biotin status in the large intestine for colonization and infection. Nat Commun 6: 6592.

Supplemental Table S2　Construction of template *recA*-mutated plasmid

Mutant plasmid	PCR primers	Template plasmid
precAG22Y1	P1079-P1080	pFRT-recA2
precAG24Y1	P1081-P1082	pFRT-recA2
precAE123A3	P1093-P1094	pFRT-recA2
precAG204S3	P1095-P1096	pFRT-recA2
precAD224A1	P1089-P1090	pFRT-recA2
precAG288Y1	P1085-P1086	pFRT-recA2

Supplemental Table S3 Construction of isogenic mutant strains

Mutant strain	PCR primers	Template plasmid	Parent strain	Target locus	Kan cassette
ERSA	P845–P846	pLCE19	EDL933	downstream of *recA*	–
E1Q1	P1064–P600	pFRT–kan	EDL933	*Q* of Stx1–phage	–
EDLf	P724–P725	pFRT–kan	EDL933	*fur*	–
ERA1–1	P10002–P845	pFRT–Kan	EDL933	*recA*	–
EP	P613–P628	pFRT–Kan	EDL933	*stx1* promotor	–
ERG22Y	P1100–P845	precAG22Y1	ERA1–1	*recA*	–
ERG24Y	P1100–P845	precAG24Y1	ERA1–1	*recA*	–
ERE123A	P1100–P845	precAG288Y1	ERA1–1	*recA*	–
ERG204S	P1100–P845	precAD224A1	ERA1–1	*recA*	–
ERD224A	P1100–P845	precAE123A3	ERA1–1	*recA*	–
ERG288Y	P1100–P845	precAG204S3	ERA1–1	*recA*	–

Table S4 Summary of the phenotypes observed for RecA variants in *recA*-deficient *E. coli* harboring mutant RecA expression plasmid and isogenic mutant *recA* EHEC strains

	UV sensitivity[a]	Recombination activity[a]	RecA upregulation[a, b]		LexA cleavage[a]	UmuD cleavage[a]	Stx2 expression[b]	NO response[b]
			a	b				
Wild	-	+++++	+	+	+	+	+	+
G22Y	++	+	+	-	-	+	+	-
G24Y	-	+	+	+	+	+	+	+
E123A	++++++	-	-	-	-		-	-
G204S	++	++++	-	-	-	-	-	-
D224A	++++	-	-	-	-		-	-
G288Y	-	+	+	+	+		+	+

[a]These results were reported from Adikesavan et al using *recA*-deficient *E. coli* strains harboring mutant RecA expression plasmid (Adikesavan et al., 2011). UV sensitivity and recombination activity were estimated by UV survival assay and P-1 transduction assay, respectively (Adikesavan et al., 2011). RecA upregulation, LexA cleavage induction and UmuD cleavage induction were analyzed by Western blotting (Adikesavan et al., 2011). The cultures of RecA-wild type or mutant *E. coli* strains were added nalidixic acid (100 μg/ml) and then incubated for 60 min at 37℃ (Adikesavan et al., 2011).

[b]These results were indicated in this study using isogenic mutant *recA* EHEC strains (ERG22Y, ERG24Y, ERE123A, ERG204S, ERD224A and ERG288Y). RecA upregulation and Stx2 production were analyzed by Western blotting. The cultures of wild-typeEHEC or mutant EHEC strains were treated with or without DETA-NONOate (200 μM) and then incubated for 18 h at 37℃ under anaerobic conditions.
